# Plants Dictate Root Microbial Composition in Hydroponics and Aquaponics

**DOI:** 10.3389/fmicb.2022.848057

**Published:** 2022-04-18

**Authors:** Victor Lobanov, Karel J. Keesman, Alyssa Joyce

**Affiliations:** ^1^Department of Marine Sciences, University of Gothenburg, Gothenburg, Sweden; ^2^Mathematical and Statistical Methods Group – Biometris, Wageningen University & Research, Wageningen, Netherlands

**Keywords:** rhizosphere, community analysis, rhizobiome, aquaponics, hydroponics

## Abstract

The role of the microbial community in mediating fish and plant co-culture is often considered the black box of aquaponics. Despite widespread recognition regarding the dependency of plants on their rhizosphere, the extent to which upstream aquaculture influences downstream hydroponic root communities has been poorly described in the literature. In this study we performed a taxonomic survey (16S rRNA metabarcoding) of microbial communities originating in the facility water source, hydroponic nutrient solution (HNS) sump, nutrient supplemented biofilter effluent (BF) sump, and recirculating aquaculture system tanks stocked with Nile tilapia (*Oreochromis niloticus*). Lettuce (*Lactuca sativa*) was then grown using the HNS and BF effluent under sterilized or mature (prior aquaponics/hydroponics lettuce culture water) conditions, likewise, the influence of probiotic addition or inoculation with soil-grown lettuce rhizosphere was assessed. Compositional similarities across treatments suggest that under soil-less conditions, plants are able to exert a stronger discriminatory influence on their rhizosphere composition than is done by colonization from upstream sources. Furthermore, cluster dendrograms grouped the sterilized and unsterilized treatments more consistently together than hydroponics and aquaponics treatments. These findings contradict conventional beliefs that microbial communities in the water column colonize roots based on their presence alone, ignoring the role that plants play in rhizosphere community selection.

## Introduction

The region in and around plant roots, the rhizosphere, is an interspecies nutrient and electron trade zone with stakeholders representing all kingdoms (Hacquard, 2017; Wang et al., 2017; Garcia and Kao-Kniffin, 2018; Geisen et al., 2018; Guyonnet et al., 2018; Hu et al., 2018). Recent studies have shown that soil-based plants exert significant pressure in terms of nutrient composition on their rooting communities (Sasse et al., 2018; Zhalnina et al., 2018; Compant et al., 2019). The extent to which these findings may be transposed onto plants grown in soil-less cultivation conditions is less clear for two reasons. Firstly, it is unclear whether the release of soluble plant exudates into an aqueous milieu diminishes their effect on the microbial community. Secondly, the greater ease by which the microbial community may be transferred within the aqueous environment could contribute to a greater capacity for root colonization.

The rhizosphere community (rhizobiome) manages nutrient uptake needs (Yang and Crowley, 2000; Scagliola et al., 2016; Garcia and Kao-Kniffin, 2018; Vadstein et al., 2018), abiotic stress resistance (Hussain et al., 2018; Sasse et al., 2018; Topalovic et al., 2020), and host defense (Gourion et al., 2015; Yasin and Ahmed, 2016; Elhady et al., 2018). It is composed of a core component fulfilling essential functions required by the plant at each stage of its growth, and a satellite component consisting of strains present at low abundances (Compant et al., 2019). The core community consists of taxa that are necessarily drawn to the root environment in contrast to bulk soil (Yeoh et al., 2017). As only 7% of bulk soil microorganisms are found in the rhizosphere (DeAngelis et al., 2009), the carbon-rich environment of the rhizosphere has been described as a precursory selection pressure. The relatively stable flow of 10–250 mg/g organic acids from the plant into the rhizosphere enriches microbial taxa two orders of magnitude greater than surrounding soil (Lynch and de Leij, 2001), with root exudates including amino acids, organic anions, sugars (Phillips et al., 2004; Badri and Vivanco, 2009; Kawasaki et al., 2016; Jacoby et al., 2020). The complex dynamics of rhizobiome development has given rise to many metagenomic studies on the rhizosphere (Kawasaki et al., 2018; Sasse et al., 2018; Vadstein et al., 2018; Ayipio et al., 2019; Compant et al., 2019). Research on soil-based studies indicates that investments into the root community is a high priority for terrestrial plants, but it is not evident how well this relationship is preserved in a nutrient solution environment such as soil-less hydroponic or integrated agriculture systems (e.g., aquaponics). Furthermore, the capacity of probiotics to mediate host plant/rhizosphere interactions was explored through the application of the commercially relevant bacterium *Bacillus amyloliquefaciens*, which has been developed as a probiotic in hydroponics but not aquaculture (Kidoglu et al., 2008; Nautiyal et al., 2013; Chowdhury et al., 2015).

In this study, a decoupled aquaponics design was used to study downstream colonization of the rhizosphere by upstream microbial communities (Goddek et al., 2019a). From a nutrient perspective, there are two inputs: fish feed for the aquaculture unit and any fertilizer addition in the hydroponics unit. Sources for microbial inoculation may arise from the local aqueous or airborne environment, as well as through the import of foreign material into the system (i.e., via feed). Recent publications focusing on the diversity of microorganisms in aquaponic systems have given rise to many hypotheses as to how the microbial community may lead to increased performance based on the increased abundance of chelating agents, cofactors, enzymes, or hormones facilitating nutrient bioavailability, either directly or indirectly (Munguia-Fragozo et al., 2015; Rurangwa and Verdegem, 2015; Schmautz et al., 2017; Sheridan et al., 2017; Kasozi et al., 2021). While the microbial community is widely recognized as important to the success of aquaponic systems (Delaide et al., 2016, 2017; Goddek et al., 2016b; Wielgosz et al., 2017; Bartelme et al., 2018; Goddek and Vermeulen, 2018; Eck et al., 2019), it has likewise been suspected as a vector for pathogen proliferation (Mori and Smith, 2019; Kasozi et al., 2021).

With the objective of determining the source of the microbial community colonizing the rhizosphere, lettuce (*Lactuca sativa*) was grown under a variety of hydroponic conditions including nutrient supplementation with a commercial hydroponic solution alone, nutrient supplemented aquaculture-derived water stocked with Nile tilapia (*Oreochromis niloticus*) and after inoculation with a probiotic or soil culture. Through multiple discriminating analyses (cluster dendrogram, principal component analysis), this study highlights the important role of plants in determining their own rhizosphere composition in soil-less cultivation systems.

## Materials and Methods

A decoupled (unidirectional flow) aquaponics system was stocked with Nile tilapia (*Oreochromis niloticus*) and Batavian lettuce (*Lactuca sativa*) Exaudio RZ 79-43 (Rijk Zwaan, Netherlands) grown at the Wageningen UR Greenhouse Horticulture Unit (Bleiswijk, Netherlands). The lettuce was grown in hydroponic boxes (3 plants/ea.) with three replicates per treatment. Boxes, insomuch as they were self-contained provided better control over microbial exposure to the plants than normal media-based, raft or nutrient film systems, but did not completely prevent bacterial transfer as growth conditions were not sterile, nor were seeds sterilized prior to planting. Each box contained a Styrofoam sheet floating on nutrient solution, mimicking a deep-water culture environment. Four microcentrifuge tubes with sheared tips were filled with 2% w/v agar-agar (Sigma, Netherlands) and inserted into the sheet with seeds immersed in the agar. Roots growing into the aqueous milieu were considered to be representative of the plants’ rhizosphere, as this most closely resembles root structure in hydroponic cultivation conditions.

For all treatments, seeds were incubated in darkness overnight (8 h) at 25°C. Filter sterilized (0.22 μm) hydroponic nutrient solution (HNS) was added to each box at the beginning of cultivation and exchanged for the treatment-specific nutrient solution after 2 days. Nutrient solutions were prepared weekly, at which time half of the volume was exchanged. Supplementation of the sump solution was done as necessary to maintain the following approximate macronutrient composition (mmol/L): 15.0 NO_3_, 1.5 NH_4_, 5.0 K, 1.5 Na, 3.0 Ca, 1.5 Mg, 0.1 Si, 0.1 Cl, 1.5 SO_4_, 0.5 HCO_3_, 0.5–1.0 P. The following trace elements set points were also maintained (μmol/l): 20.0 Fe, 7.0 Mn, 5.0 Zn, 20.0 B, 0.5 Cu, 0.1 Mo, while pH (set to 6–7) and EC (set to 2–2.5 mS/cm) were adjusted as needed to maintain desired ranges. Studies directly comparing yields between aquaponics and hydroponics have proven difficult to reproduce (Ayipio et al., 2019; Yep and Zheng, 2019). As most aquaponic and hydroponic systems strive to maximize crop productivity through the same conventional means (greenhouse design, cultivar selection, etc.), nutrient concentrations were kept constant in this study to avoid confounding the relationship between nutrient loading and plant health.

**FIGURE 1:**
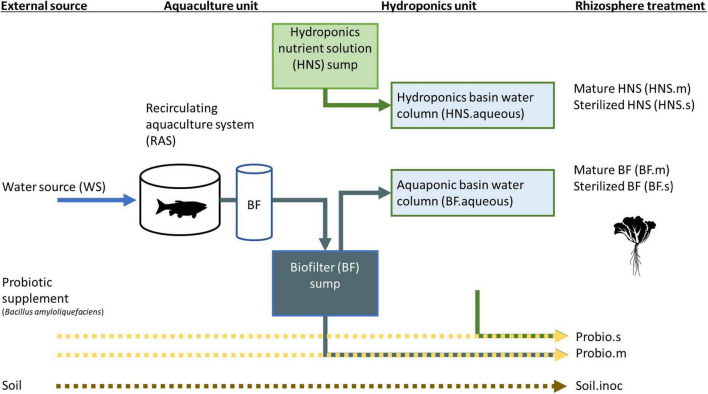
Summary of treatments in the current study.

Treatments were watered from either the aquaponics system (BF) or a commercial hydroponic nutrient solution (HNS) (Figure 1). Aquaponics crops received effluent from the biofilter, with nutrient supplementation carried out in a decantation tank prior to the hydroponics unit. Here, we refer to HNS from two full crop cycle as mature HNS (HNS.m). To make sterilized HNS (HNS.s) or BF (BF.s), freshly made nutrient stock solutions were filter sterilized (0.22 μm). The probiotic effect of *B. amyloliquefaciens* was added to sterilized HNS and to unsterilized BF (corresponding to treatments Probio.s and Probio.m, respectively). A DSMZ (Germany) culture stock of *B. amyloliquefaciens* (ex Fukumoto 1943) grown in pure culture to 5 × 10^11^ CFU/g stock was applied to achieve a final concentration of 2 mg/L. Soil inoculum (ca. 50 mg) was sourced from Batavian lettuce grown in potting soil for 4 weeks. The soil sample was sequenced as a control (referred to as “Soil”), inoculated treatments are referred to as Soil.inoc. The water column from Batavian lettuce grown aquaponic (BF.aqueous) and hydroponic (HNS.aqueous) basins were furthermore sampled as a control for the pelagic microbial community, as was the facility water source (WS) and the aquaculture tanks (RAS).

Water samples during all three trials were analyzed weekly for nutrient concentrations, pH, and EC (Groen Agro Control, Netherlands). Dissolved oxygen (DO) was kept saturated for both experiments. Temperature was controlled at 16°C. Broad spectrum lighting was maintained at 200 μmol/s/m^2^ for 16 h/day for all trials, although supplemental lighting was not used for trial 2 (due to summer conditions providing adequate irradiation). Crops were harvested after 6 weeks.

For microbial community profiling, DNA was isolated from the roots of each technical replicate using the DNeasy PowerSoil Kit (Qiagen, Germany). All plant roots in an individual box (technical replicate) were combined for DNA extraction. Roots were lightly shaken but not directly dried so as not to influence the rhizosphere community prior placement inside the microcentrifuge tube used in the protocol. A noticeable film of water enveloped the roots after shaking; 0.25 g of wet roots were used for the DNA extraction. The PowerSoil kit was chosen as it is well adapted to extract DNA from complex matrices such as the extracellular polymeric substances consistent with biofilm structure. For soil samples (soil inoculum referred to above as “Soil”), 0.25 g of soil from around the root was used. Purified DNA was PCR amplified using universal 16S rDNA bacterial primers (Table 1) targeting the V3-V4 region of the 16S rRNA gene. Primers were provided by BaseClear B.V. (Netherlands) and sequenced using their MiSeq system. Sequenced operational taxonomic units (OTUs) were processed as per BaseClear protocols whereby sequenced amplicons are merged into overlapping pseudo-reads and subsequently aligned against the NCBI 16S rRNA database for putative taxonomic identification.

**TABLE 1:** Primer sequences used for the taxonomic community analysis in this study.

Domain target	Direction	Sequence	Length (bp)	Melting temperature	GC%
Bacteria	Fwd primer	AGAGTTTGATCCTGGCTCAG	20	56.92	50.00
	Rv primer	ATTACCGCGGCTGCTGG	17	60.18	64.71

In R, the OTU data set was subdivided into six data frames related to the taxonomic rank using the Tidyverse packages tidyr and dplyr. Subsequent analyses were restricted to the genus, family, and order ranks as a compromise between the large amount of OTUs generated in the data set (obscuring clear visualization of the data) and to avoid a lack of resolution as occurring at higher ranks. Firstly, vegan was used for the diversity analysis, ade4 and labdsv were used for multivariate data analyses, pvclust for hierarchical cluster analysis, vegclust and vegsoup for data clustering, picante for community analysis, and finally corrplot for the correlation plot. Packages for visualization of the data included gclus to generate the clustering graphics, dendextend for dendrograms, and ggplot2 for the correlation plot.

Due to the effect of outliers, several normalization strategies were explored: presence/absence, maximum abundance per treatment, relative abundance per species, relative frequency per site, normalization to the Euclidian norm (Chord transformation), normalization to the relative frequency per site (Hellinger transformation), double profile normalization (Chi-squared transformation), and normalization first by species maxima then by site totals (Wisconsin standardization). Normalization by Hellinger transformation were chosen for this study based on the tightness of the variance range in the processed data sets.

Three types of neighbor clustering were used to organize the data: nearest, furthest, and Ward. Nearest neighbor clustering agglomerates groups based on the shortest pairwise dissimilarities between members, while the furthest neighbor method defines the group membership based on the maximum distance between any two clusters. Ward’s minimum variance clustering minimizes the total within-cluster variance and appeared the most logical to follow based on the robustness of the groups. The optimal number of clusters were calculated using Ward correlation, Pearson correlation, IndVal method, simple structure index (ssi) criteria, and Calinski criteria (Dolnicar et al., 2000; Borcard et al., 2018). While the range of optima was fairly consistent across taxonomic ranks, the optimization algorithms never converged on a single figure. The clustering result was then independently confirmed by a principal component analysis and correlation plot of the treatments. Finally, network analyses of both the treatments and microbial taxa allowed us to visualize which treatments most closely resemble each other at different taxonomic ranks.

## Results

In terms of plant health, treatments were not nutrient limited nor displayed signs of disease. Lacking obvious indications of stress, it was assumed that plants interacted with the surrounding microbial environment under homogenous circumstances across treatments with differences in community composition originating from the source water and not physicochemical or stress factors. To elucidate the relationship between the host plants and the composition of the rhizosphere microbial community, this study investigated patterns in taxonomic prevalence across treatments through hierarchal classification and clustering analyses.

**FIGURE 2:**
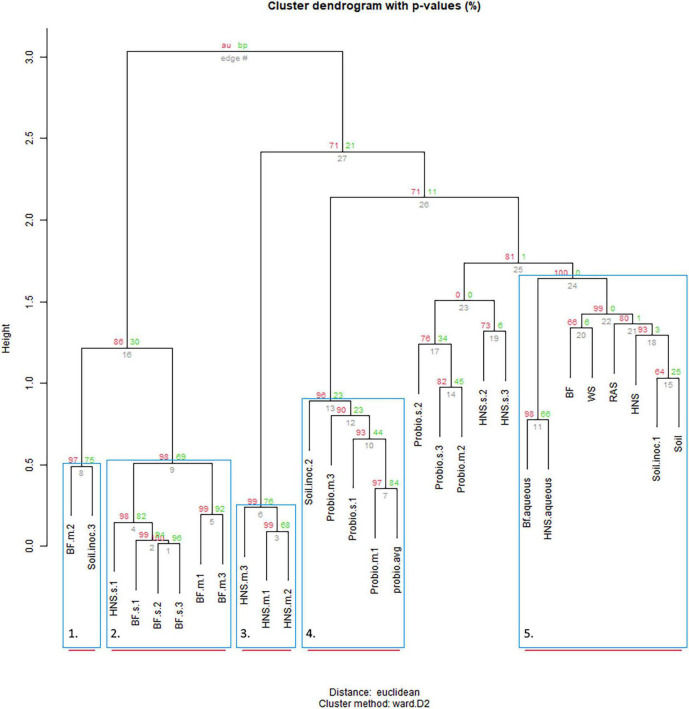
Cluster dendrogram of the distribution of microbial communities at the genus rank across treatments with the five most robust clades highlighted. Similar patterns were observed at higher ranks. Treatments include hydroponic nutrient solution sump (HNS) and biofilter effluent sump (BF) under mature (.m), sterilized (.s), and basin water column (.aqueous) conditions. Additionally, soil inoculum (Soil) and HNS inoculated culture (soil) and probiotic (probio) inoculated sterilized (.s) and unsterilized biofilter effluent (BF) samples, as well as the facility water source (WS) and recirculating aquaculture system water column (RAS) are also included.

Plotting the distribution of OTUs across the treatments provided evidence for the existence of a core microbiome present in many (9–10 out of 28 treatments), although most OTUs are unique to 1–2 treatments (Figure 2); plots of the family and order rank (Supplementary Figures 1, 2) were similar but not identical. Approximate unbiased (red, significant ≥0.95) and bootstrap probability (green, value indicates the amount of bootstrapping until robust) *p*-values for the edge dendrograms (edge number in gray) are indicated. Blue squares indicate significance at *p* ≥ 0.90 with red bars indicating high robustness at *p* ≥ 0.95. At the genus rank it is visible that the source of colonization does not strongly predict clustering. Aquaculture derived water appears to influence community dynamics whether sterilization is imposed (BF.s) or not (BF.m) (group 2), however, mature BF or HNS box communities (group 3) were not closely related to the aqueous community used to inoculate the boxes. The control treatments (BF, WS, HNS, RAS, Soil) cluster similarly (group 4), with the aqueous communities (aquaculture linked or independent) clustering closely together. Probiotic supplementation mostly clustered in group 5, however, some branches were mixed with other treatments. At the family rank (Supplementary Figure 1) no clear pattern was visible, although it is visible that the probiotic treatments populated one branch at the first fork, while the controls and most mature and sterilized HNS treatments populated the second fork.

**FIGURE 3:**
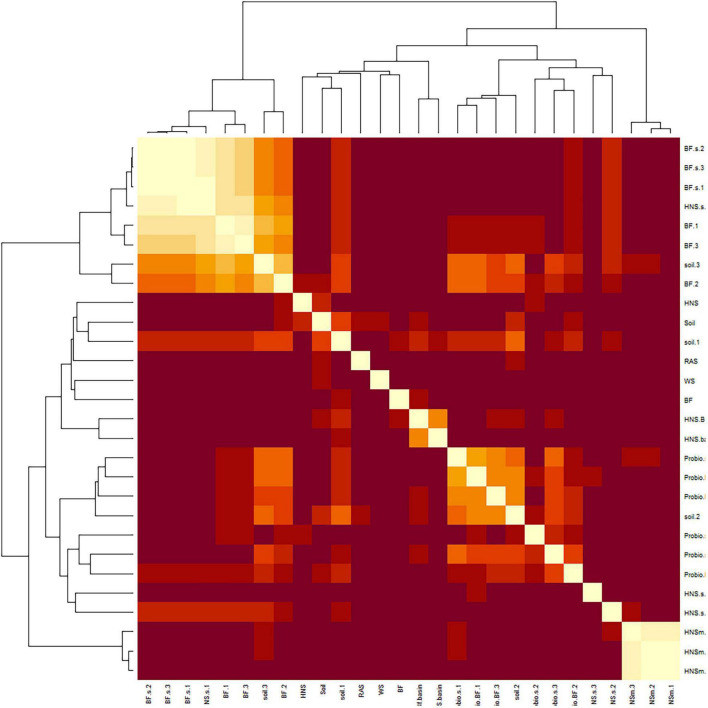
Dissimilatory matrix between microbial communities of the treatments in the study at the genus rank. Similar patterns were observed at higher ranks. Treatments include hydroponic nutrient solution sump (HNS) and biofilter effluent sump (BF) under mature (.m), sterilized (.s), and basin water column (.aqueous) conditions. Additionally, soil inoculum (Soil) and HNS inoculated culture (soil) and probiotic (probio) inoculated sterilized (.s) and unsterilized biofilter effluent (BF) samples, as well as the facility water source (WS) and recirculating aquaculture system water column (RAS) are also included.

**FIGURE 4:**
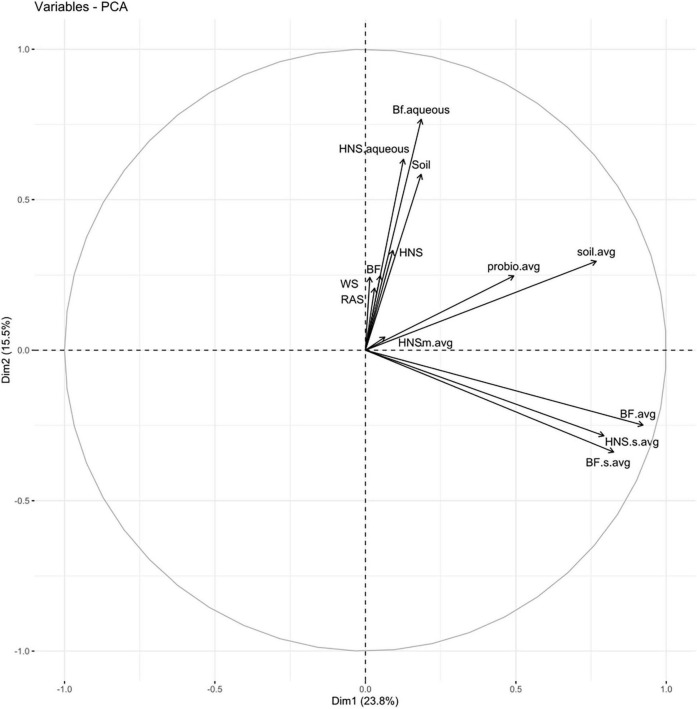
Principle component analysis for all treatments; abundance data across technical replicates were averaged for each set. Treatments include hydroponic nutrient solution sump (HNS) and biofilter effluent sump (BF) averaged for each technical replicate and basin water column (.aqueous) conditions. Additionally, soil inoculum (Soil) and HNS inoculated culture (soil) and probiotic (probio) samples averaged for all technical replicates, as well as the facility water source (WS) and recirculating aquaculture system water column (RAS) are also included.

Partitioning based on ssi criteria resulted in multiple equally optimal partitions for a range of cluster objects. Ultimately, this indicates a high degree of interchangeability between most treatments, suggesting that the microbial communities present are more similar than different. Looking at a dissimilatory matrix of the treatments (Figure 3), we see that the aquaculture impacted (BF series) and probiotic supplemented (Probio) treatments tend to be more similar within themselves that to each other, with the soil and standard hydroponics (HNS series) being less cohesive groups. The principle component analysis (Figure 4) places the mature HNS treatments (HNS.m) at the center of the distribution, with the two most discriminating factors at 23.8% (dimension 1) and 15.5% (dimension 2) causing a split between the controls (WS, RAS, BF, HNS, Soil, HNS.aqueous, and BF.aqueous) and experimental treatments (HNS.m, HNS.s, BF.m, and BF.s). The probiotic (probio) and soil inoculated (soil) treatments were less dependent on the two principal dimensions.

**FIGURE 5:**
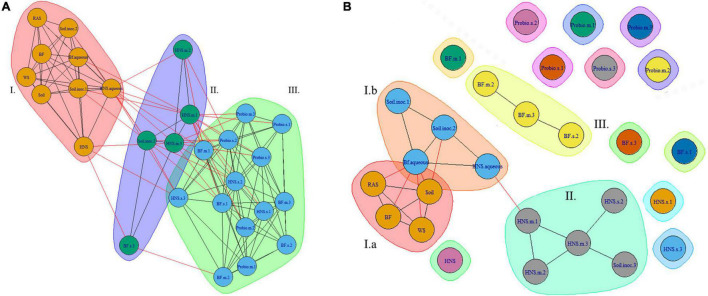
Co-occurrence network of microbial communities across treatments at the class **(A)** and family **(B)** ranks. Treatments include hydroponic nutrient solution sump (HNS) and biofilter effluent sump (BF) under mature (.m), sterilized (.s), and basin water column (.aqueous) conditions. Additionally, soil inoculum (Soil) and HNS inoculated culture (soil) and probiotic (probio) inoculated sterilized (.s) and unsterilized biofilter effluent (BF) samples, as well as the facility water source (WS) and recirculating aquaculture system water column (RAS) are also included.

Finally, co-occurrence networks were generated for microbial communities. At higher ranks the superstructure for community similarity across treatments is more clearly defined. At the order rank this appears as three clusters, two of which are more closely related (Figure 5A). At lower ranks (Figure 5B) these clusters begin to splinter as the quantity of unique labels corresponding to microbial taxa increases exponentially.

## Discussion

This study is the first to investigate how the rhizosphere microbial community is shaped by upstream influences under soil-less cultivation conditions. Lettuce (*Lactuca sativa*) was grown hydroponically or in aquaponics co-culture with Nile tilapia (*Oreochromis niloticus*). As shown in Figure 1, treatments included nutrient supplementation with a commercial hydroponic solution alone, nutrient-supplemented aquaculture-derived water, or the commercial nutrient solution inoculated with a probiotic or soil culture. Filter sterilization vs. inoculation with mature media (nutrient solution derived from a previously harvested lettuce culture) were tested for both hydroponic and aquaponic treatments as well as the probiotic addition.

As indicated by the cluster dendrogram (Figure 2), no divisive split grouping all aquaponic (BF.m and BF.s) apart from all commercial hydroponic (HNS.m and HNS.s) treatments exists at the genus rank despite a highly robust clustering model with a cophenetic correlation of 0.93, a pattern consistent across different clustering methods and at higher taxonomic ranks. The number of optimal clusters, however, varied from 2 to 9 clusters between the five methods tested, mirroring the overall dendrogram shape when viewed based on cluster height. After an initial branching into 2–4 groups, the height difference between clusters drops sharply – reflecting a higher degree of replaceability. Either as part of a main or sub-branch, the controls (BF, WS, RAS, HNS, and Soil) tended to cluster closely. These controls mainly serve to identify environmentally prevalent microorganisms from the water supply (WS), aquaculture unit (RAS), nutrient-supplemented biofilter effluent sump (BF), hydroponic nutrient solution sump (HNS), and local soil-based lettuce rhizosphere (Soil). Their high degree of similarity at low taxonomic ranks suggests that most microorganisms are ubiquitously present, in agreement with the rare biosphere ecological model (Lynch and Neufeld, 2015; Jousset et al., 2017).

Another perspective of community similarity is portrayed in the dissimilatory matrix (Figure 3), comparing treatments by virtue of their degree of similarity instead of being clustered based on a threshold consensus as well as the co-occurrence networks (Figures 5A,B), where clustering is allowed to overlap if treatments are sufficiently similar. At the class rank (Figure 5A), an agglomeration of the aquaponics treatments (3 BF.s, 2 BF.m) is visible in cluster III. The BF control and BF water column samples were distinct from this group (cluster I), sharing a greater degree of similarity with the other controls instead. While the mature HNS treatments (HNS.m, cluster II) clustered together, their cluster partially overlapped with cluster III containing the three HNS.s treatments. The probiotic treatments clustered together (cluster III), however, the soil treatments were distributed between clusters I and II. Much of the cluster similarity disappeared at the genus rank, albeit the nodes of clusters I and II are still visible (Figure 5B).

From the PCA (Figure 4) and the co-occurrence network analysis at the genus rank (Figure 5B), it is visible that the probiotic treatments poorly grouped together on average, meaning that their taxonomic composition was the least consistent within technical replicates. One may speculate that the colonizing influence of the probiotic shifted the community as a whole, rearranging the rhizobiome into a different configuration than in other treatments. Insofar as this may be attributed to the probiotic itself is outside the scope of this study.

Soil treatments did not consistently cluster together (Figures 2, 4, 5), ostensibly reflecting the shift in community composition from bulk soil to the rhizosphere environment as described elsewhere (DeAngelis et al., 2009). Community diversity was poorly retained when soil-based lettuce roots were used to inoculate sterile HNS. These treatments gravitated toward the same global consensus as the other hydroponic treatments rather than forming a robust branch independently, despite filter sterilization of the HNS and no direct contact between media. Although a portion of the HNS sump microbiome is shared with HNS.m and HNS.s treatments, several taxa undergo major shifts in abundance during this transition. As a model this suggests that under similar nutrient concentrations, rhizosphere involvement plays a greater role in driving microbial community composition than water source.

An array of factors influencing rhizobiome composition have been identified, originating both from the plant (genotype, life stage) and the environment (water source, nutrient profile) (Compant et al., 2019; Chen et al., 2020). As shown by Bartelme et al. (2019), facility conditions strongly dictate the microbial populations present in RAS and aquaponic systems. Our results suggest that a similar facility-specific microbiome forms within the rhizosphere in hydroponic systems. Studies on the rhizobiome in other type of cultivation systems such as soil or air have indicated a similar pattern of consolidation. For instance, Schreiter et al. (2014) observed that the lettuce rhizobiome was consistent across varying soil types, while Edmonds et al. (2020) observed a rhizobiome unique from the circulating nutrient solution that formed after 12 days of plant growth in aeroponic conditions (Edmonds et al., 2020). This trend appears to be a hallmark of terrestrial plants (Berg et al., 2014; Vandenkoornhuyse et al., 2015; Sasse et al., 2018; Zhalnina et al., 2018). In combination with the results from this study, it appears that selection pressures exerted by the plant to consolidate the rhizobiome around a particular profile are a fundamental aspect of plant physiology despite the influence of the exogenous microbial environment. That profile, although observed as a collection of taxa, mirrors the functional needs required by the plant at a particular life stage and under particular environmental conditions.

At a more global level, microbial communities will occupy all available niches as they become available. For instance, among its many discoveries, the Tara Oceans project revealed that physiochemical parameters such as pH and temperature play a more decisive role in the relative taxonomic abundance than does taxonomic presence (Gorsky et al., 2019; Sunagawa et al., 2020). Co-occurrence networks (Supplementary Figure 3) at the family and order rank indicate consistent grouping of certain microbial clades. However, further research should combine our top-down approach with a bottom-up strategies to study community organization [e.g., identification of keystone species (Herren and McMahon, 2018)], as well as omics based techniques for community functional analysis, to elucidate how select microorganisms or clades may impact facility productivity through their disproportionate influence on community structure.

Understanding the potential impact of upstream microbial communities on downstream hydroponic units has direct implications for preventative disease management. Demonstrating that the rhizosphere community composition is associated with the plant more strongly than the presence of exogenous colonizing bacteria implies that focusing efforts on supporting plant health rather than on water sterilization will better protect crops. Sterilization of incoming water and media is widely used in hydroponics to discourage the proliferation of pathogens (Ehret et al., 2001; Shimizu et al., 2007; Raudales et al., 2014; Liu and Huang, 2019; Zheng et al., 2019) albeit at the cost of reducing overall microbial diversity – both beneficial and harmful microorganisms – potentially opening niches for rapid colonization by r-strategists (Vadstein et al., 2018).

Some aquaponic studies advocate for continuous cycling of water between RAS and HP components (coupled aquaponics) (Nichols and Savidov, 2011; Palm et al., 2019), while others have advocated for a discrete separation (decoupled aquaponics) with no return of water and hence microorganisms from the HP to the RAS (Delaide et al., 2016; Goddek et al., 2016a,2019b; Goddek and Keesman, 2018; Goddek and Korner, 2019; Monsees et al., 2019). In this context, we sought to determine whether sterilization (reducing microbial proliferation across units) succeeds in significantly shaping the microbial community structure. Clustering did not indicate a mature/sterilization split at the genus (Figure 2), family, or order ranks (Supplementary Figures 1, 2), nor was a strong split visible via the dissimilatory matrix (Figure 3). Most treatments furthermore clustered together at the class rank (Figure 5A), with the notable exception being the mature HNS treatments in cluster II. In a prior investigation into the effect of sterilization in the context of RAS coupling, Wielgosz et al. (2017) concluded that the beneficial effects on plant growth from RAS effluent were most likely conferred through microbial exudates, and thus unaffected by the sterilization process itself. While the identity of those exudates remains unknown, our results further support their hypothesis by showing that the community composition is not principally determined by the source water (HNS/BF) or source community (mature/sterilized).

In terms of microbial compositional diversity, the most profound shift occurred between controls and treatments in a stepwise manner (Supplementary Figure 3). The soil control indicated a high level of diversity with a couple phyla disappearing in soil-inoculated treatments (*Fibrobacteres, Nitrospinae*), however, the majority of phyla were present at reduced concentrations. The facility water supply control (WS) was relatively enriched with some phyla compared to the recirculating aquaculture system (RAS) and biofilter (BF): *Bacillariophyta*, *Chlamydiae, Aquificae, Candidatus Saccharibacteria*. The RAS and BF conditions enriched the phyla *Fusobacteria, Nitrospirae*, and *Lentisphaerae*. Few members of these phyla could be detected in subsequent aqueous (BF.aqueous) or rhizosphere (BF.1-3) environments suggesting a lack of viability in the oligotrophic, ammonia-poor, hydroponic environment. Probiotic treatments (Probio.m.1-3, Probio.s.1-3) most significantly perturbed the total microbial composition. While no mechanism could be determined within the context of this study, probiotics have been shown to restrict microbial diversity in the gut microbiome (Suez et al., 2018). Regardless, further studies must corroborate our results to elucidate the relationship between probiotic administration and community diversity. In terms of co-occurrence, no clear patterns of taxonomic clustering could be discerned. At high taxonomic ranks, the amount of overlap consolidates most taxa together while at lower ranks the diversity creates an unmanageable number of sub-groups. At the order rank, some discernable clustering is visible (Supplementary Figure 4), however, the significance of these co-occurrences could not be determined within the scope of this study.

Our study focused on the microbial community dynamics at the main interface between the aqueous milieu and the plant in soil-less cultivation systems – the rhizosphere. The above trends indicate community consolidation in our system, suggesting that prioritizing plant health metrics will likewise reduce the potential for disease. We have recently demonstrated that trace nutrients are not taken up by plants proportionally to their external aqueous concentrations (Lobanov et al., 2021), which suggests that fundamental issues such as plant nutritional needs should be prioritized. Given the slow growth requirements of k-strategists [e.g., anammox (Laureni et al., 2015; Zhang et al., 2017; Zielińska et al., 2018), archaea (Seyler et al., 2014; Bartelme et al., 2017)], system-wide maturation of the microbial population may take months or years (Savidov and Brooks, 2004). It would not be unreasonable to expect successive waves of colonization to mark this period, as is similarly observed within the rhizobiome during plant growth (Micallef et al., 2009; Kristin and Miranda, 2013; Chaparro et al., 2014). Archaea and eukaryotic phyla (algae) were observed in the study at the phylum rank (Supplementary Figure 4), however, their contribution to rhizosphere structure, organization, and nutrient flow in aqueous environments remains an open question. While not investigated here, community succession in the rhizobiome during facility maturation may indicate the duration within which a facility microbiome stabilizes and thus is able to maximally resist pathogen colonization.

## Conclusion

In this study, we have provided evidence that plant crop health is poorly predicted by exposure to upstream microbial communities in soil-less aquaponic cultivation systems. This study is the first to address the question of rhizosphere-colonizing microbial transfer in aquaponics by selectively exposing hydroponically grown plants to a range of treatments intended to shape the root microbiome. Prior literature has suggested that upstream aquaculture directly contributes to crop productivity through microbial colonization (Bartelme et al., 2018; Eck et al., 2019), or in other cases, may represent an entry point for pathogens into the system (Mori and Smith, 2019; Kasozi et al., 2021). While our data do not exclude these possibilities, they instead suggest that the introduction of upstream bacteria is less impactful than previously assumed. More likely, plant health weaknesses are exploited by pathogenic microorganisms ubiquitous in the local environment, thus not uniquely introduced through the water column. We expect the findings of this study to be transferable to cultivation conditions where healthy plants are not subject to excessive stress (i.e., due to nutrient deficiency or other water quality perturbations), however, future research must investigate how these systems respond to acute abiotic or biotic stressors.

This work paves the way for two important future directions. Firstly, our study suggests that aqueous nutrient concentration play a more predicative role in determining community composition than sterilization. While sterilization is a routine technique in aquaculture as well as hydroponics, it is nonetheless a tradeoff between pathogen suppression and total microbial diversity reduction. Future studies must likewise determine whether aquaponic facilities benefit from sterilization, or whether the co-cultivation of plants and fish in an environment promoting diversity leads to a more resilient facility-wide microbiome. Secondly, in line with previous work on the relationship between aqueous nutrient concentrations and plant health (Lobanov et al., 2021), more research is needed to determine whether a greater focus on maintaining plant health as opposed to only maximizing yield will lead to more disease-tolerant crops, and ultimately more productive crops.

## Data Availability Statement

The datasets presented in this study can be found in online repositories. The names of the repository/repositories and accession number(s) can be found below: https://www.mg-rast.org/mgmain.html?mgpage=project&project=mgp101035, mgm4954329.3.

## Author Contributions

AJ and VL conceived the presented idea. VL carried out the experiments and wrote the manuscript with input from all authors.

## Conflict of Interest

The authors declare that the research was conducted in the absence of any commercial or financial relationships that could be construed as a potential conflict of interest.

## Publisher’s Note

All claims expressed in this article are solely those of the authors and do not necessarily represent those of their affiliated organizations, or those of the publisher, the editors and the reviewers. Any product that may be evaluated in this article, or claim that may be made by its manufacturer, is not guaranteed or endorsed by the publisher.

## Supplementary Material

Supplementary Figure 1 Cluster dendrogram of the distribution of microbial communities at the order rank across treatments with the five most robust clades highlighted. Similar patterns were observed at higher ranks. Treatments include hydroponic nutrient solution sump (HNS) and biofilter effluent sump (BF) under mature (.m), sterilized (.s), and basin water column (.aqueous) conditions. Additionally, soil inoculum (Soil) and HNS inoculated culture (soil) and probiotic (probio) inoculated sterilized (.s) and unsterilized biofilter effluent (BF) samples, as well as the facility water source (WS) and recirculating aquaculture system water column (RAS) are also included.

Supplementary Figure 2 Cluster dendrogram of the distribution of microbial communities at the family rank across treatments with the five most robust clades highlighted. Similar patterns were observed at higher ranks. Treatments include hydroponic nutrient solution sump (HNS) and biofilter effluent sump (BF) under mature (.m), sterilized (.s), and basin water column (.aqueous) conditions. Additionally, soil inoculum (Soil) and HNS inoculated culture (soil) and probiotic (probio) inoculated sterilized (.s) and unsterilized biofilter effluent (BF) samples, as well as the facility water source (WS) and recirculating aquaculture system water column (RAS) are also included.

Supplementary Figure 3 Distribution of phyla across treatments and controls.

Supplementary Figure 4 Co-occurrence network of microbial taxa at the order rank across treatments.
