# Supplementary figures and images for: Plants Dictate Root Microbial Composition in Hydroponics and Aquaponics
Source: Front Microbiol. 2022 Apr 18;13:848057. doi: 10.3389/fmicb.2022.848057 (PMC9058158; doi:10.3389/fmicb.2022.848057)

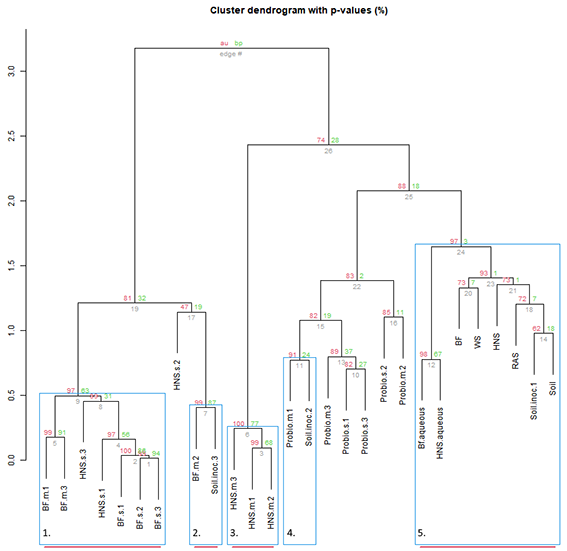

Supplement: Supplementary Figure 2 — Cluster dendrogram of the distribution of microbial communities at the family rank across treatments with the five most robust clades highlighted. Similar patterns were observed at higher ranks. Treatments include hydroponic nutrient solution sump (HNS) and biofilter effluent sump (BF) under mature (.m), sterilized (.s), and basin water column (.aqueous) conditions. Additionally, soil inoculum (Soil) and HNS inoculated culture (soil) and probiotic (probio) inoculated sterilized (.s) and unsterilized biofilter effluent (BF) samples, as well as the facility water source (WS) and recirculating aquaculture system water column (RAS) are also included. [file Image_2.TIFF]

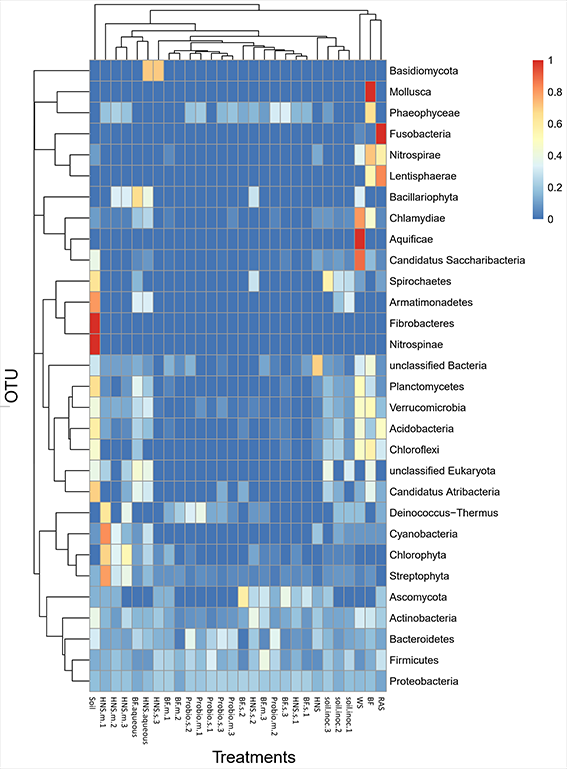

Supplement: Supplementary Figure 3 — Distribution of phyla across treatments and controls. [file Image_3.TIFF]

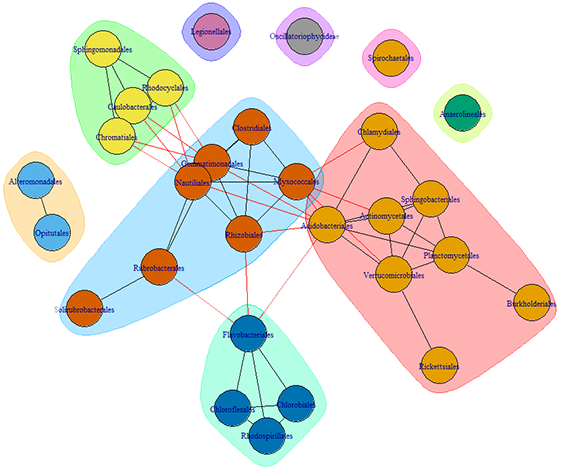

Supplement: Supplementary Figure 4 — Co-occurrence network of microbial taxa at the order rank across treatments. [file Image_4.tiff]
